# Personalized brain MRI revealed distinct functional and anatomical disruptions in Creutzfeldt‐Jakob disease and Alzheimer's disease

**DOI:** 10.1111/cns.14404

**Published:** 2023-08-14

**Authors:** Yanjun Guo, Jianxun Ren, Weigang Cui, Louisa Dahmani, Danhong Wang, Xiaoxuan Fu, Meiling Li, Shiyi Li, Yi Zhang, Xue Lin, Zhen Zhen, Yichen Xu, Dan Xie, Hongzhi Guan, Fang Yi, Jiawei Wang, Qi Shi, Hesheng Liu

**Affiliations:** ^1^ Department of Neurology Beijing Tongren Hospital, Capital Medical University Beijing China; ^2^ Changping Laboratory Beijing China; ^3^ School of Engineering Medicine Beihang University Beijing China; ^4^ Department of Radiology Athinoula A. Martinos Center for Biomedical Imaging, Massachusetts General Hospital, Harvard Medical School Charlestown Massachusetts USA; ^5^ Department of Radiology Beijing Friendship Hospital, Capital Medical University Beijing China; ^6^ Department of Neurosurgery Beijing Tiantan Hospital, Capital Medical University Beijing China; ^7^ Department of Neurology Beijing Friendship Hospital, Capital Medical University Beijing China; ^8^ Department of Neurology Peking Union Medical College Hospital, Chinese Academy of Medical Sciences Beijing China; ^9^ Department of Neurology Lishilu Outpatient, Jingzhong Medical District, Chinese PLA General Hospital Beijing China; ^10^ State Key Laboratory for Infectious Disease Prevention and Control, National Institute for Viral Disease Control and Prevention Chinese Center for Disease Control and Prevention Beijing China; ^11^ Biomedical Pioneering Innovation Center Peking University Beijing China

**Keywords:** Alzheimer's disease, brain cortical thickness, Creutzfeldt‐Jakob disease, fMRI

## Abstract

**Aims:**

Creutzfeldt‐Jakob disease (CJD) is a lethal neurodegenerative disorder, which leads to a rapidly progressive dementia. This study aimed to examine the cortical alterations in CJD, changes in these brain characteristics over time, and the differences between CJD and Alzheimer's disease (AD) that show similar clinical manifestations.

**Methods:**

To obtain reliable, subject‐specific functional measures, we acquired 24 min of resting‐state fMRI data from each subject. We applied an individual‐based approach to characterize the functional brain organization of 10 patients with CJD, 8 matched patients with AD, and 8 normal controls. We measured cortical atrophy as well as disruption in resting‐state functional connectivity (rsFC) and then investigated longitudinal brain changes in a subset of CJD patients.

**Results:**

CJD was associated with widespread cortical thinning and weakened rsFC. Compared with AD, CJD showed distinct atrophy patterns and greater disruptions in rsFC. Moreover, the longitudinal data demonstrated that the progressive cortical thinning and disruption in rsFC mainly affected the association rather than the primary cortex in CJD.

**Conclusions:**

CJD shows unique anatomical and functional disruptions in the cerebral cortex, distinct from AD. Rapid progression of CJD affects both the cortical thickness and rsFC in the association cortex.

## Graphical abstract

Patients with Creutzfeldt‐Jakob disease (CJD) show less atrophy in the primary cortex than patients with Alzheimer's disease (AD). Patients with CJD show more severe functional disruption than patients with AD.

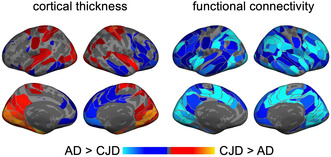

## 1 INTRODUCTION

Human prion diseases are rare and lethal neurodegenerative disorders. Creutzfeldt‐Jakob disease (CJD) is the most common human prion disease with less than 1‐year survival time from the disease onset.^1^, ^2^ Rapidly progressive dementia is the hallmark of CJD, along with executive deficits, aphasia, and gait difficulty.^3^ Similar to CJD, Alzheimer's disease (AD), the most common neurodegenerative disorder, manifests with progressive dementia and various cognitive deficits. However, AD dementia progression usually lasts around 5 years.^4^ CJD and AD share similar clinical signs but present vastly different progressions.^5^ Previous studies have compared cerebrospinal fluid proteins,^6^ abnormal intensities of brain imaging,^7^ and electroencephalography^5^ of CJD and AD patients to clinically differentiate the two diseases. However, the direct comparison of the anatomical and functional brain differences between the two diseases is still lacking. This is partly due to the short survival time of CJD, which leaves a very narrow time window to investigate the brain changes over time. Unveiling the anatomical and functional brain disruptions in CJD may shed light on the neural underpinnings of the rapid disease progression.

MRI was extensively applied in clinical diagnosis of CJD.^7^, ^8^ The hyperintensities in diffusion‐weighted imaging (DWI) and fluid‐attenuated inversion recovery signals, as biomarkers, show high sensitivity and specificity for discriminating CJD from controls.^9^, ^10^ DWI studies revealed white matter abnormalities in CJD.^11^, ^12^ Progressive brain atrophy was also associated with the deterioration of clinical symptoms in CJD.^13^ However, diffusive and structural MRI modalities cannot capture brain functional changes. An effective tool for examining brain function is resting‐state functional MRI (rsfMRI), which has been widely used in brain disorder research. rsfMRI estimates resting‐state functional connectivity (rsFC) in the brain. In AD patients, studies indicate the disruption in rsFC mainly affecting the default mode network (DMN), which is associated with the memory loss.^14^ To date, however, rsfMRI has rarely been applied to CJD research.^15^

To elucidate anatomical and functional disruption in CJD and difference between AD and healthy controls, we collected structural MRI and rsfMRI data in 10 CJD patients and eight well‐matched AD patients and eight healthy controls. We measured and compared rsFC strength and cortical thickness within personalized functional regions to control for the substantial interindividual variability.^16^ Moreover, we scanned a subset of the CJD patients (*n* = 5) twice and delineated their progressive functional disruption and cortical atrophy patterns.

## 2 MATERIALS AND METHODS

### 2.1 Participants

**FIGURE 1:**
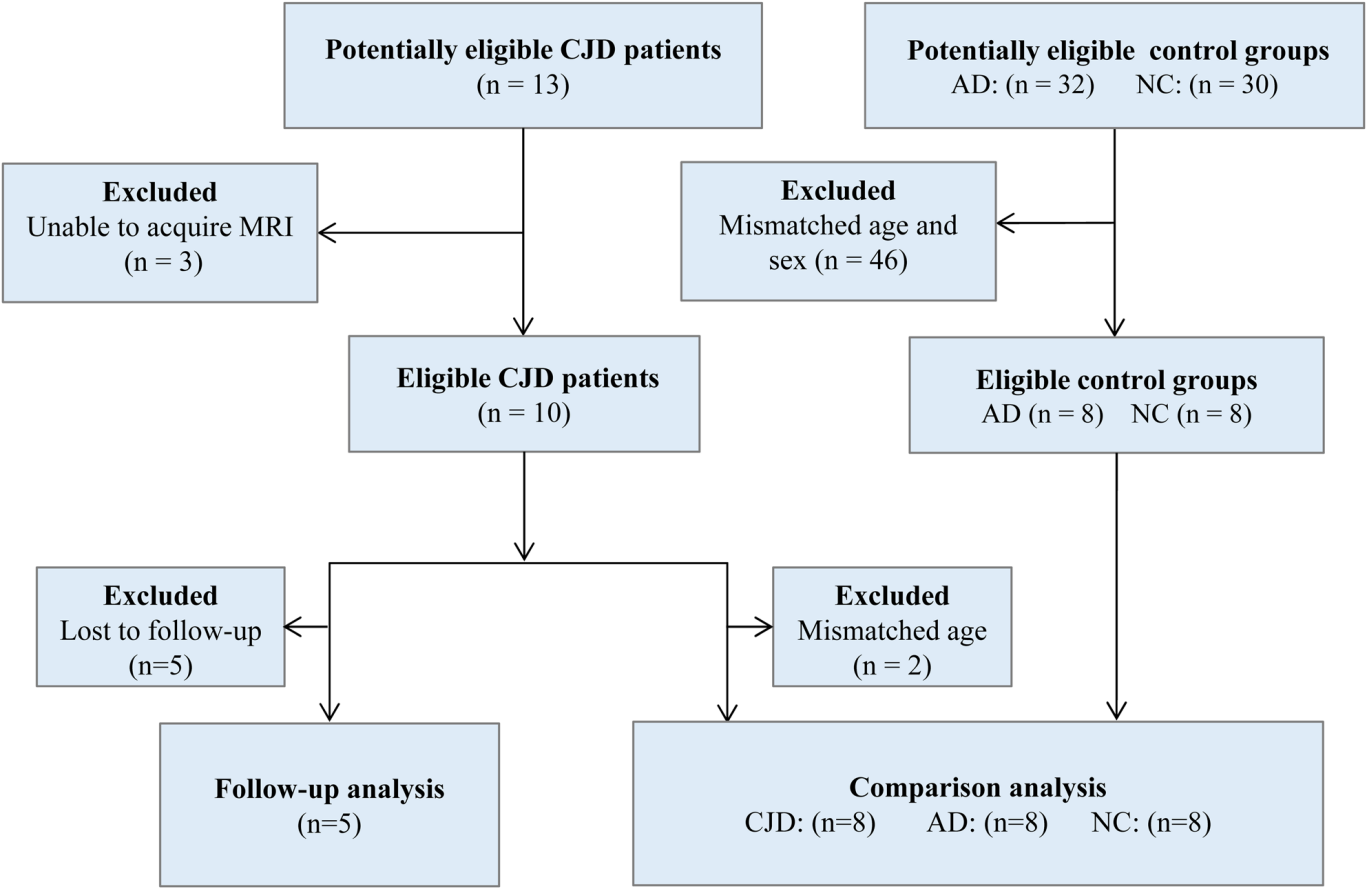
Study flowchart shows the recruitment of CJD patients, AD patients, and normal controls. AD, Alzheimer's disease; CJD, Creutzfeldt‐Jakob disease.

All participants or their designees provided informed consent, which was approved by the Research Ethics Committee of Capital Medical University. We screened 13 patients who had a neurologist‐confirmed diagnosis of probable or definite CJD from 2019 to 2020 (Table 1). Three patients were excluded due to MRI contraindications. Each of the included 10 patients underwent a high‐resolution anatomical scan and four 6‐min BOLD rsfMRI scans (24 min in total) at each time point to examine the anatomical and functional features of the cerebral cortex. Moreover, five of them underwent additional scans with intervals of 41–221 days (average interval, 120.4 ± 75.5 days) to examine the longitudinal brain changes. To compare CJD patients with healthy participants and AD patients, we chose eight normal control (NC) participants and eight patients with severe AD with matched age and gender to each CJD patient, respectively. Of note, two patients with CJD were too young to be matched with AD patients (Table 1, CJD04 is 38 years old and CJD05 is 43 years old). Hence, we excluded them in the comparisons between NC and AD, but included them in the longitudinal analyses. Figure 1 shows the patient selection and subject matching flowchart.

**TABLE 1:** Clinical and neuroradiological characteristics of patients with CJD.

	CJD01	CJD02	CJD03	CJD04	CJD05	CJD06	CJD07	CJD08	CJD09	CJD10
Age (years)/sex	49/M	65/M	51/M	38/M	43/F	72/M	60/F	64/F	56/F	62/F
Disease duration (month)	14	8	4	24	3	15	4	2	2	1
Education (years)	15	9	12	6	16	12	12	5	12	12
Scan interval (days)	221	41	102	64	174	/	/	/	/	/
DWI hyperintensity										
Cerebral cortex	+	+	+	+	+	+	+	+	+	+
Striatum	+	−	−	+	−	−	+	−	+	−
Thalamus	−	−	−	+	+	−	−	−	−	−
Cerebellum	−	−	−	−	−	+	−	−	−	−
PRNP codon 129	M/M	M/M	M/M	M/M	M/M	M/M	M/M	M/M	M/M	M/M
PRNP mutation				G114V	G114V		T88K			
14‐3‐3 protein	−	−	+	+	−	+	−	−	−	−
MMSE baseline	26	5	20	15	24	17	12	20	9	10
MMSE follow‐up	10	/	9	21	1	/	/	/	/	/
CDR‐SB baseline	5	16	10	6	5	11	9	2	8	8
CDR‐SB follow‐up	10	/	12	6	/	/	/	/	/	/
MoCA baseline	20	1	10	9	/	10	5	14	/	/
MoCA follow‐up	6	/	5	17	/	/	/	/	/	/

Abbreviations: CDR‐SB, clinical dementia rating sum of boxes scores; DWI, diffusion‐weighted imaging; F, female; M, male; MMSE, Mini‐Mental State examination; MoCA, Montreal Cognitive Assessment; PRNP, prion protein gene.

### 2.2 Data collection and data preprocessing

Each patient underwent anatomical and functional MRI scans on a Siemens Prisma 3T scanner (Siemens Healthcare) equipped with a 64‐channel head coil. Structural images were obtained using a MP‐RAGE 3D T1‐weighted sequence (TR = 5000 ms; TE = 2.98 ms; flip angle = 0°; 1.0 mm isotropic voxels; FOV = 256 × 240). Functional MRI (fMRI) data were acquired using an echo planar imaging (EPI) pulse sequence (TR = 3000 ms; TE = 30 ms; flip angle = 85°; 3.00 × 3.00 × 3.75 mm^3^ voxels, matrix size = 72 × 72, 47 slices).

It has been repeatedly shown that reliability of resting‐state functional connectivity measures is a function of scan length.^17^, ^18^ To obtain reliable functional measures from each patient, subjects underwent four 6‐min resting‐state scan (24 min total) wherein the patient was instructed to stay awake and keep their eyes open. The resting‐state fMRI and structural MRI data were preprocessed using the same procedures as described in our previous reports^19^, ^20^, ^21^ and Supplementary Methods.

### 2.3 Estimation of rsFC

The individual‐specific rsFC between seed regions of interest (ROI) was estimated by calculating Pearson correlations between each seed ROI's average time series and those of other ROIs. Correlation values were converted to *z* values using Fisher's *r*‐to‐*z* transformation.

### 2.4 Individual‐specific fine‐grained functional parcellation

To control for the substantial intersubject variability in functional connectivity, we identified 213 functionally personalized cortical regions for each individual.^22^ The fine‐grained parcellation contains 108 regions in the left hemisphere and 105 regions in the right hemisphere. First, a population‐level fine‐grained parcellation was created based on a sample of 1000 young healthy participants.^23^ Second, we applied an iterative clustering approach that was adjusted from our previously reported method^22^, ^24^, ^25^ to generate individual‐specific mapping in each region, and the details are given in Supplementary Methods. To perform a network analysis, we assigned each of the fine‐grained ROIs to one of the eight canonical large‐scale functional networks (Figure S1).

### 2.5 Estimation and comparison of cortical thickness and rsFC

The vertex‐wise cortical thickness was estimated by FreeSurfer.^26^ Cortical thickness was averaged within each individual‐specific ROI. To compare cortical thickness between groups, for each ROI we averaged the thickness within each group. We also quantified the difference in cortical thickness at the network level, using the eight canonical networks as well as the primary/association cortices.

To estimate ROI‐wise rsFC, for each ROI, we averaged the rsFC between the ROI and other ROIs within the same functional network. The individual‐specific ROI‐wise rsFC maps were averaged across participants from a group. The difference between groups was further quantified in eight networks as well as in the primary/association cortices.

### 2.6 Statistical analysis

Before performing statistical comparisons between groups, covariates including age, head motion, and temporal signal noise ratio (tSNR) were regressed from the cortical thickness and functional connectivity. The Wilcoxon rank‐sum test was used to compare metrics (cortical thickness and rsFC) between two different groups, due to the small sample size. The Wilcoxon signed‐rank test was used to statistically compare the longitudinal change in five CJD patients across two separate scans. Multiple comparisons for the eight‐network analysis were corrected using the Bonferroni correction.

## 3 RESULTS

### 3.1 Patient characteristics

The study included 10 patients with CJD (mean age, 56 ± 11 years; 5 women). Demographic and clinical information of patients is provided in Table 1. To compare CJD with AD and healthy controls, eight age‐matched patients with AD (mean age, 61 ± 8 years; 4 women) and eight NC participants (mean age, 60 ± 8 years; 4 women) were included. No significant difference in demographics, including gender, age, and education, and in fMRI data quality, including head motion, and tSNR, was detected in the three groups (Table 2). The Mini‐Mental State Examination (MMSE) was performed to assess global cognition. The CJD and AD groups did not show a significant difference in global cognition (CJD, 14.88 ± 7.02, AD, 16.50 ± 3.16, Wilcoxon rank‐sum test, *p* = 0.36) but significantly poorer global cognition than the NC participants (CJD, 14.88 ± 7.02, NC, 28.00 ± 1.51, Wilcoxon rank‐sum test, *p* < 0.001).

**TABLE 2:** Characteristics and fMRI data qualities of CJD, AD, and healthy controls.

Parameters	CJD	AD	NC	*χ* ^2^	*p*‐Value
	Mean ± SD	Mean ± SD	Mean ± SD		
Characteristics					
Gender	4/4	4/4	4/4	0	1*
Age (years)	59.88 ± 7.62	60.74 ± 7.61	60.00 ± 7.89	0.16	0.922**
Education (years)	10.38 ± 3.42	10.25 ± 5.80	12.38 ± 3.29	0.98	0.614**
MMSE	14.88 ± 7.02	16.50 ± 3.16	28.00 ± 1.51	15.08	0.0005**
fMRI data quality					
FD (mm)	0.11 ± 0.10	0.13 ± 0.06	0.10 ± 0.03	2.02	0.365**
tSNR	230.57 ± 93.36	163.03 ± 75.20	226.45 ± 47.46	4.33	0.115**

Abbreviations: FD, framewise displacement; MMSE, Mini‐Mental State Examination; NC, normal control; SD, standard deviation; tSNR, temporal signal‐to‐noise ratio.

* *χ* ^2^ test.

** Kruskal–Wallis test.

### 3.2 CJD patients show severe cortical atrophy that is distinct from AD patients

**FIGURE 2:**
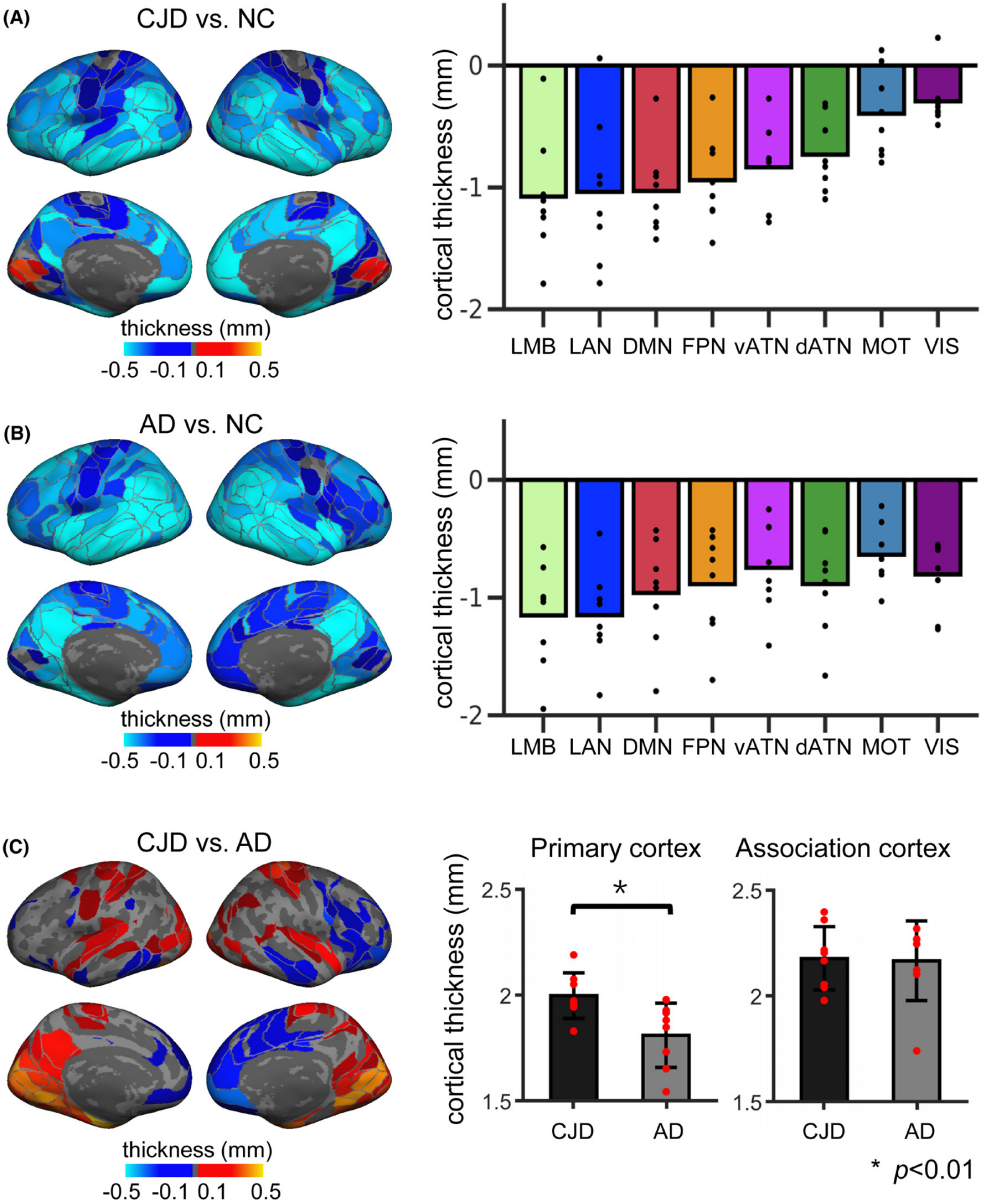
CJD shows severe cortical atrophy and distinct atrophy patterns from AD. To control for the substantial intersubject variability in functional network organization and to enhance the functional homogeneity of functional cortical regions, we localized 213 functionally homologous ROIs for each individual. (A) The cortical surface maps (left) show cortical atrophy in the patient groups assessed by comparing their cortical thickness to that in a healthy cohort. Cool colors indicate cortical thinning in CJD, with light blue indicating more severe cortical atrophy. Warm colors represent greater cortical thickness in CJD compared with NC. The cortical maps show a pattern of severe cortical atrophy in the association cortex in CJD and more modest thinning in the primary cortex. The bar graph (right) shows cortical thinning (patient cortical thickness—NC cortical thickness) averaged within each of the eight large‐scale networks. Each black dot represents cortical thinning for one CJD patient. All networks show cortical atrophy. Networks in the association cortex show greater cortical thinning than those in the primary networks (*p* = 0.008, Wilcoxon signed‐rank test). (B) AD patients' cortical thickness was contrasted against that of NC. The cortical maps (left) show a pattern of severe cortical atrophy in the posterior cortex and in the temporal lobes and less severe atrophy in the frontal lobe. Average cortical thinning (right) shows important cortical atrophy for AD patients in all networks. (C) We compared the cortical thickness of the CJD and AD groups (left). Warm colors represent thicker cortex in CJD, and cool colors represent thinner cortex in CJD. The contrast map shows that the primary cortex and medial temporal lobe are thicker in CJD than in AD, while a few frontal and temporal ROIs, especially in the right hemisphere, are thinner in CJD. We compared CJD and AD's cortical thickness in the primary cortex and association cortex (right). AD shows thinner cortex in the primary cortex (*p* = 0.006, Wilcoxon rank‐sum test), while no significant difference was found in the association cortex (*p* = 0.40, Wilcoxon rank‐sum test). Each red dot represents cortical thickness for one CJD or AD patient. AD, Alzheimer's disease; CJD, Creutzfeldt‐Jakob disease; dATN, dorsal attention network; DMN, default mode network; FPN, frontal–parietal network; LAN, language network; LMB, limbic network; MOT, motor network; vATN, ventral attention network; VIS, visual network.

We localized 213 functionally homologous ROIs in each individual and compared CJD, AD, and NC on their cortical thickness and functional connectivity. Compared with NC, CJD patients show evident cortical thinning in regions widely distributed across the cerebral cortex, with the exception of the VIS (Figure 2A, left). At the network level, CJD patients exhibit significantly greater cortical thinning in all eight functional networks than NC (Figure 2A, right; all *p*'s < 0.006, Wilcoxon rank‐sum test, Bonferroni correction). Intriguingly, our results show that CJD more severely affects cortical thickness in the association cortex (−0.94 ± 0.39 mm) rather than the primary cortex (−0.34 ± 0.27 mm, *p* < 0.001, Wilcoxon signed‐rank test).

Next, we performed parallel analyses in the patients with AD to characterize their cortical atrophy compared with NC. Similar to CJD, patients with AD show widespread cortical thinning on the whole cortex (Figure 2B, all *p*'s < 0.006 for all networks, Wilcoxon rank‐sum test, Bonferroni correction). Furthermore, to directly compare cortical atrophy patterns in CJD and AD, we contrasted the cortical thickness of patients with CJD against that of patients with AD (Figure 2C). CJD patients exhibit thinner cortex in the lateral temporal lobe, lateral and medial prefrontal cortex mostly in the right hemisphere compared with AD patients (blue ROIs in Figure 2C, left). On the contrary, AD patients show thinner cortex mainly in the primary cortex as well as the medial temporal lobe, compared with CJD (red ROIs in Figure 2C, left). We further compared CJD and AD patients' cortical thickness in primary cortex and association cortex (Figure 2C, right). There was a significant difference in the primary cortex, with CJD patients exhibiting greater cortical thickness than AD patients (CJD, 2.00 ± 0.11 mm, AD, 1.81 ± 0.15 mm, *p* = 0.006, Wilcoxon rank‐sum test), while we found no significant difference in the association cortex (CJD, 2.18 ± 0.15 mm, AD, 2.17 ± 0.19 mm, *p* = 0.40).

### 3.3 CJD patients show disruptions of rsFC compared with NC and AD patients

**FIGURE 3:**
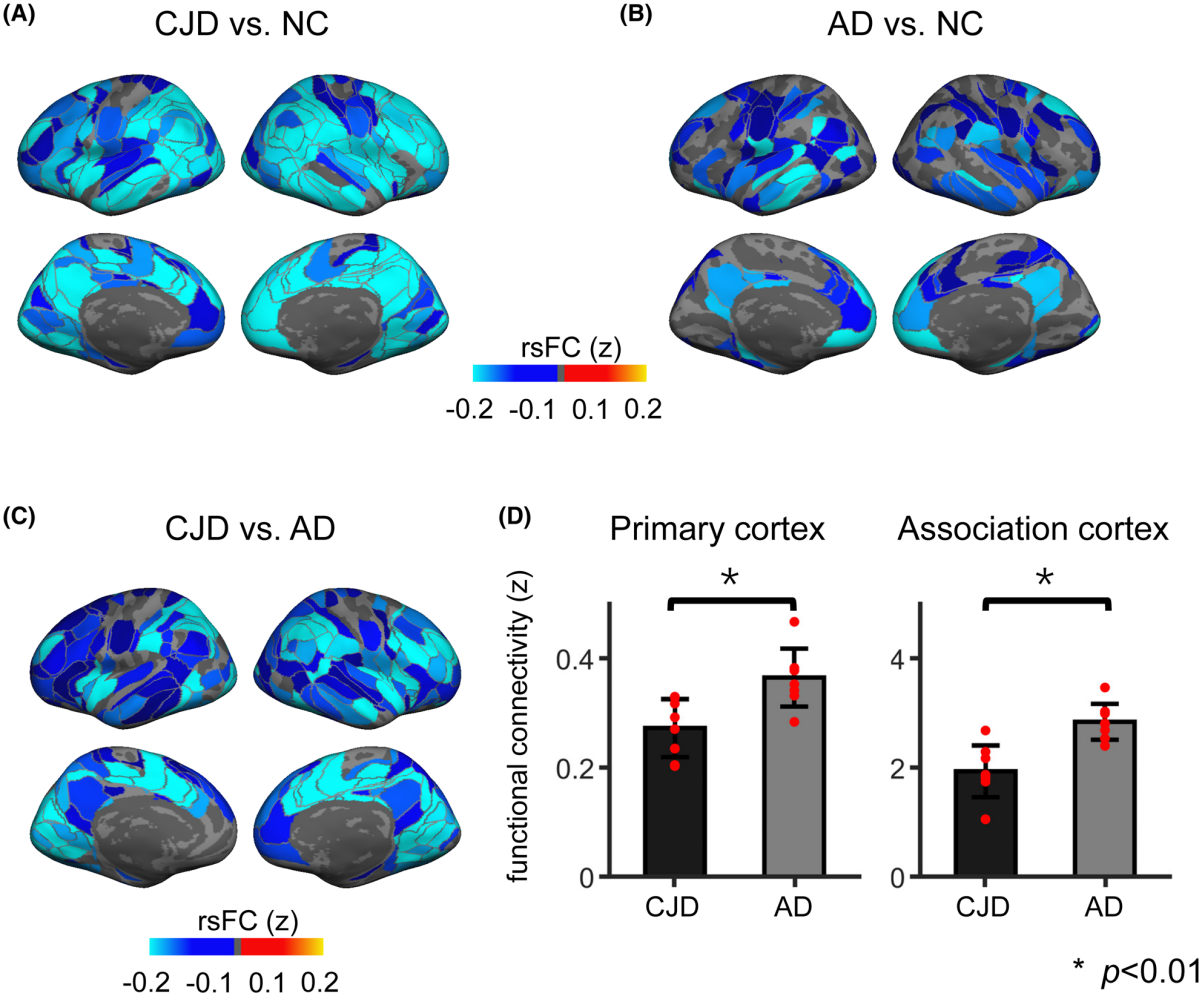
Functional connectivity disruption in CJD. (A) rsFC strength was contrasted between CJD and NC. Cool colors represent ROIs with weaker rsFC strength in CJD. The cortical maps show widespread functional connectivity disruption in CJD. (B) Compared with NC, functional disruption in AD patients is mainly observed within the DMN. (C) The rsFC strength of CJD and AD was directly contrasted. Cool colors represent ROIs with weaker rsFC strength in CJD. The maps show globally weaker rsFC strength in CJD. (D) RsFC strength is significantly weaker in CJD than AD in both the primary cortex (*p* = 0.006, Wilcoxon rank‐sum test) and the association cortex (*p* < 0.001, Wilcoxon rank‐sum test), suggestive of more severe functional disruptions in CJD than AD. Each red dot represents rsFC strength for one CJD or AD patient. AD, Alzheimer's disease; CJD, Creutzfeldt‐Jakob disease; DMN, default mode network; NC, normal control; ROI, regions of interests; rsFC, resting‐state functional connectivity; NC, normal control; ROI, regions of interests.

We estimated and averaged the within‐network rsFC strength of each individual‐specific fine‐grained ROI. We controlled confounds that substantially impact rsfMRI data quality, including head motion and tSNR. We first contrasted CJD against NC on their rsFC strength (Figure 3A). CJD patients exhibit a wide pattern of rsFC strength disruption across the whole cortex, as compared to NC. Apart from the limbic network, the rsFC strength within all functional networks was consistently lower in CJD patients (Figure S2A, Wilcoxon rank‐sum test, *p*'s < 0.006 for the seven networks excluding the LMB network, Bonferroni correction). The disruption pattern in rsFC partially overlapped with the cortical atrophy pattern (Dice Coefficient of top half of disrupted ROIs = 53.7%), but is nonetheless spatially distinct from the atrophy pattern (Spearman correlation, *r* = 0.055, *p* = 0.42). Although patients with AD also demonstrate widespread reduction in rsFC strength, the reduction is mainly concentrated in the DMN (Figure 3B; Figure S2B, Wilcoxon rank‐sum test, *p* < 0.006 for DMN, Bonferroni correction). Compared with AD, CJD demonstrates more severe disruption of rsFC in all functional networks, including the DMN (Figure 3C; Figure S2C, Wilcoxon rank‐sum test, *p*'s < 0.006 for seven networks apart from LMB, Bonferroni correction). CJD patients show weaker rsFC strength than AD patients in both the primary cortex (CJD, 0.27 ± 0.05, AD, 0.36 ± 0.05, Wilcoxon rank‐sum test, *p* = 0.006) and the association cortex (CJD, 0.19 ± 0.05, AD, 0.28 ± 0.03, *p* < 0.001, Figure 3D), suggesting that, at equal levels of global cognition, there is more severe, global functional disruption of the brain in CJD patients than AD patients.

### 3.4 Longitudinal anatomical and functional alterations in CJD mainly affect the association cortex

**FIGURE 4:**
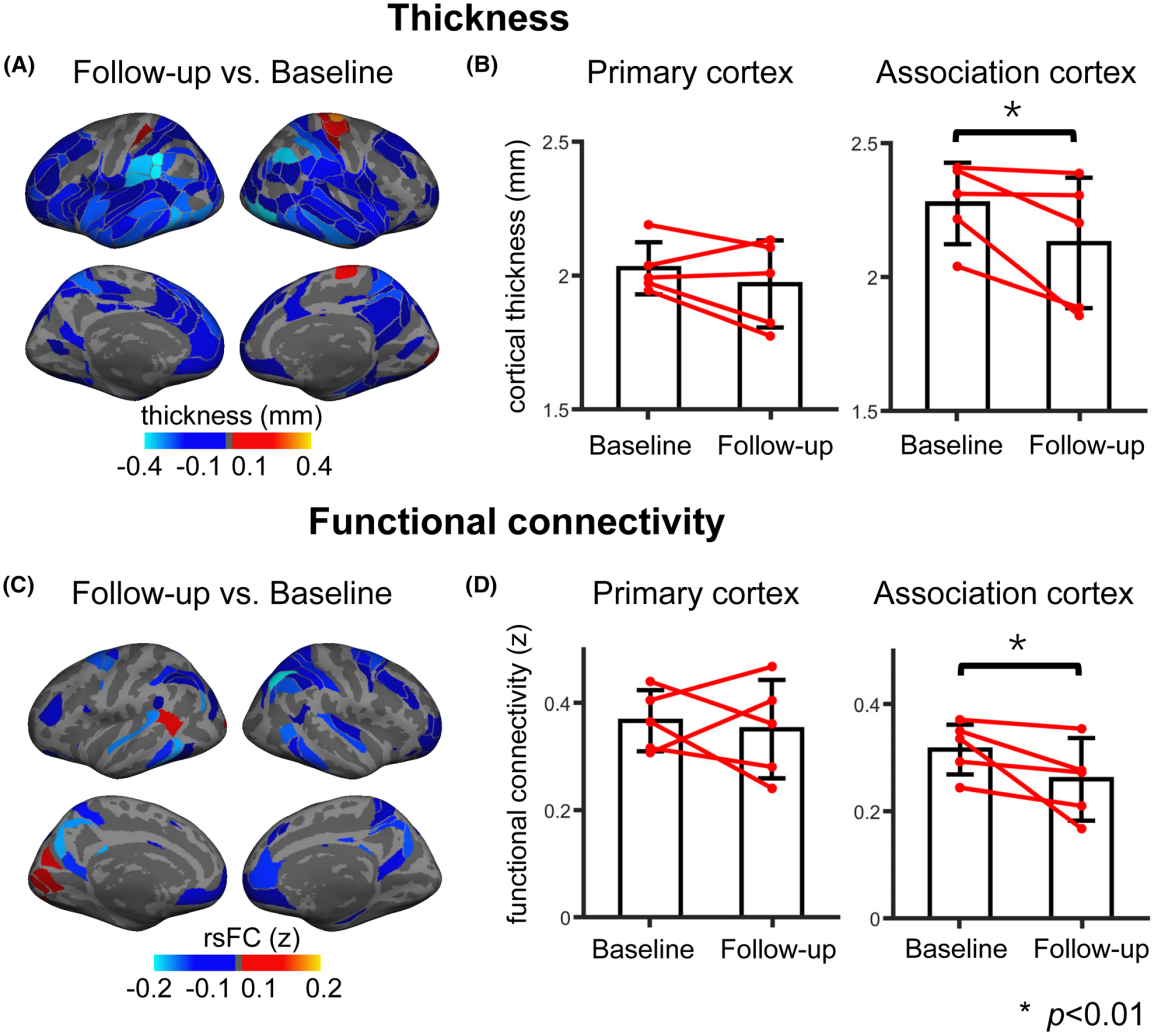
Progressive anatomical and functional changes mainly affect the association cortex. (A) In five CJD patients who had longitudinal scans, cortical thickness was reduced at follow‐up compared with baseline visit in many association areas. Blue colors represent thinner ROIs at follow‐up, while warm colors represent thicker regions of interests (ROIs) at follow‐up. (B) At follow‐up, CJD patients exhibit significantly thinner cortex in the association cortex (*p* = 0.008, Wilcoxon signed‐rank test), but no significant difference in the primary cortex (*p* = 0.68, Wilcoxon signed‐rank test). Each pair of red dots represents cortical thickness for one CJD patient. (C) rsFC strength at follow‐up was reduced in some association areas. Cool colors represent ROIs with weaker rsFC strength at follow‐up, while warm colors represent ROIs with stronger rsFC strength at follow‐up. (D) The second scan shows significantly weaker rsFC strength in the association cortex (*p* = 0.008, Wilcoxon signed‐rank test), but no significant difference in the primary cortex (*p* = 0.68, Wilcoxon signed‐rank test), indicating that CJD progressively affects the association cortex. Each pair of red dots represents rsFC strength for one CJD patient. AD, Alzheimer's disease; CJD, Creutzfeldt‐Jakob disease; ROI, regions of interests; rsFC, resting‐state functional connectivity.

We performed longitudinal analyses by investigating cortical thickness and rsFC strength in five CJD patients who underwent two separate scans. Cortical thinning occurred across the association cortex and was most pronounced in the inferior parietal lobule and inferior lateral temporal cortex (Figure 4A). Among the large‐scale functional networks, all exhibited significant cortical thinning across visits, with the exception of the motor cortex (Figure S3A, Wilcoxon signed‐rank test, *p*'s < 0.006, Bonferroni correction). In the primary cortex, slight, but nonsignificant cortical thinning was found between the two scans (Figure 4B, baseline, 2.03 ± 0.10 mm, follow‐up, 1.97 ± 0.16 mm, Wilcoxon signed‐rank test, *p* = 0.68). In the association cortex, cortical thickness was significantly reduced in the second scan (Figure 4B, baseline, 2.27 ± 0.15 mm, follow‐up, 2.13 ± 0.24 mm, Wilcoxon signed‐rank test, *p* = 0.008). Significant disruption in rsFC strength was also found at follow‐up and was sparsely distributed in the FPN, dATN, and DMN (Figure S3B; Figure 4C, Wilcoxon signed‐rank test, *p*'s < 0.006 for FPN, dATN, and DMN, Bonferroni correction). RsFC strength significantly decreased in the association cortex (baseline, 0.32 ± 0.05, follow‐up, 0.26 ± 0.08, Wilcoxon signed‐rank test, *p* = 0.008) but not in the primary cortex (Figure 4D, baseline, 0.37 ± 0.06, follow‐up, 0.35 ± 0.09, Wilcoxon signed‐rank test, *p* = 0.68). To illustrate the change in rsFC strength across time, we placed two seeds, one in the DMN and one in the MOT, and generated their group‐averaged rsFC maps. The MOT seed shows slight differences in rsFC between the two scans, while the DMN seed shows obvious rsFC reductions at follow‐up (Figure S4). Moreover, the reduction in FC rather than the cortical thinning shows modest association with time intervals (see Figure S5 and Supplementary Results). Interestingly, the CJD04 patient was an atypical case, demonstrating an increase in cognitive ability (Table 1; MMSE: baseline = 15, follow‐up = 21; MoCA: baseline = 9, follow‐up = 17). This improvement was accompanied by preserved cortical thickness in both the primary and association cortices. Additionally, the patient exhibited maintained rsFC strength in the association cortex, along with a mild increase in rsFC strength in the primary cortex during the follow‐up period (see Figure S6 and further elaboration in the discussion section).

## 4 DISCUSSION

Here, we investigated the anatomical and functional brain alterations that occur in CJD, and compared them to those in AD and NC. We found that patients with CJD have severe cortical atrophy compared with healthy control participants and that their pattern of cortical atrophy is distinct from that of patients with AD. Patients with CJD also show a significantly more severe reduction in rsFC strength across almost the whole cerebral cortex compared with patients with AD, despite controlling for global cognition. Moreover, the longitudinal changes in both cortical thickness and rsFC strength in CJD were mainly associated with the association cortex rather than the primary cortex.

Our results show that cortical atrophy in CJD mainly affects the association functional networks, such as the limbic, default mode, and attention networks, which encompass the frontal, parietal, and temporal lobes. A similar atrophy pattern was revealed in a voxel‐based morphometry study that involved 24 CJD patients with early‐stage disease.^13^ Interestingly, the pattern of cortical atrophy in CJD is remarkably similar to the pattern of hyperintensities in DWI.^8^, ^27^ The consistent spatial distribution of disruptions observed in different imaging modalities may stem from CJD's pathophysiology. The spongiform feature associated with nerve cell swelling and extracellular space invading is characteristic to the CJD, thereby impacting the mean diffusivity of the affected brain areas and resulting in the appearance of hyperintensities in DWI.^28^, ^29^ An advanced degree of neuronal and cytoarchitectural loss, along with severe spongiform change, may lead to cortical atrophy.^28^, ^29^

Of note, the disruption in rsFC in CJD is evenly distributed among almost all association networks, including the ATN, DMN, FPN, and LAN, which is consistent with the findings from DWI studies that show the cerebral cortex is widespread and early affected.^30^ The functional disruptions and structural atrophy observed in the cerebral cortex likely contribute to a general disruption of brain function and may account for the nonspecific clinical symptoms observed in CJD. This finding holds the potential promise in combining functional and structural MRI to obtain more targeted imaging endpoints for future clinical trials.

Moreover, the pattern of disruption observed in CJD is distinct from the predominant disruption of the DMN in AD. Despite both diseases presenting with similar clinical signs of dementia, CJD and AD exhibit distinct patterns of disease progression and impairments in cognitive domains.^5^ This functional discrepancy may also explain the differing cognitive impairments these two diseases present. In CJD, there is a severe and global cognitive decline, including memory loss, executive control dysfunction, and speaking difficulty,^31^ while memory loss is the predominant symptom in AD. Investigating these differences may aid in understanding the underlying etiology of CJD and in selecting more appropriate therapeutic options for this rapidly progressive disease.

The cross‐sectional comparisons in cortical thickness and rsFC with a healthy cohort show that CJD mainly affects the association cortex. The longitudinal comparisons corroborate this finding, with disruptions in rsFC and cortical thinning occurring mainly in the association cortex over time. Cortical thinning was mostly observed in the supramarginal gyrus, insular, and inferior temporal cortex. Similar patterns of cortical thinning were shown in patients with inherited human prion disease and patients with multiple‐source CJD.^13^, ^32^ One study showed that only left‐insular cortical atrophy was significantly correlated with the cognitive changes in CJD.^13^ Different from the widely distributed cortical thinning we observed in our CJD patients, rsFC disruption was mainly concentrated in the FPN, dATN, and DMN. The disruption of these networks may be associated with the impairments in executive function, attention, and memory.^31^ Although both cortical thinning and disrupted rsFC seemed to mainly affect the association network, the spatial distribution of these patterns was nonetheless different, with a modest spatial correlation between the two. This modest correlation indicates that functional disruptions may be partially dependent on anatomical substrates.

Furthermore, objective evaluation of disease progression is crucial but lacking in CJD treatment trials.^33^ Our results show that rsFC changes rather than anatomical changes are modestly associated with time between assessments. This association implicates that rsFC is sensitive to disease progression and that rsFC may be an additional, objective outcome measure in treatment clinical trials. Therefore, fMRI may be a useful candidate tool in the evaluation of therapeutic effectiveness as well as in the monitoring of disease progression.

Intriguingly, we observed an atypical case (CJD04) that displayed remarkable cognitive enhancement across multiple assessments within a relatively short interval of 6 weeks. Notably, the rsFC and cortical thickness of the association network exhibited minimal changes over time in this patient. CJD04 carried the G114V mutation and was diagnosed with familial CJD, a type of disease known for its relatively slow progression.^34^ Furthermore, the patient received targeted cognitive training administered by family members and a daily medication regimen consisting of 10 mg of donepezil and 10 mg twice daily of memantine. These factors likely contributed collectively to the observed cognitive improvement within a relatively short timeframe. This intriguing case of atypical cognitive enhancement in CJD patients holds the potential promise of specific cognitive training interventions and pharmacological treatments in the context of delaying disease progression in CJD patients.^35^, ^36^

Two limitations are worthy of note in the study. First, the sample size of CJD patients was limited due to the rarity. Although the sample size is too small to draw strong conclusions, significant differences were still found between different diseases as well as longitudinally. The fact that we found significant differences may be a reflection of the severity of CJD and of its rapid progression. It is also possible that long scans and the individual‐specific method to identify functionally homologous ROIs in participants helped to obtain more precise measurements of brain organization and may have in part counteracted the heterogeneity introduced through functional misalignment in this limited sample size.^37^ Second, all patients with probable diagnosis of CJD in this study were MM129, which is the most prevalent in China. Thus, these results can only be applied to subtypes MM1, MM2, or mixed MM1 + 2 rather than other subtypes.

In conclusion, CJD shows unique anatomical and functional disruptions in the cerebral cortex. Rapid progression of CJD affects both the cortical thickness and rsFC in the association cortex. These findings may improve our understanding of the disease's pathophysiology.

## AUTHOR CONTRIBUTIONS

**Yanjun Guo, Jianxun Ren, and Hesheng Liu:** Contributed to the conception and design of the study. **Weigang Cui, Jianxun Ren, Xiaoxuan Fu, Meiling Li, Yi Zhang, Xue Lin, Zhen Zhen, Yichen Xu, Dan Xie, Hongzhi Guan, Fang Yi, Jiawei Wang, and Qi Shi:** Contributed to the acquisition and analysis of data. **Yanjun Guo, Jianxun Ren, and Weigang Cui:** Verified the underlying data. **Yanjun Guo, Jianxun Ren, Weigang Cui, Louisa Dahmani, Danhong Wang, Shiyi Li, and Hesheng Liu:** Contributed to drafting the text and preparing the figures. **Yanjun Guo, Jianxun Ren, and Weigang Cui:** Contributed equally to this work. All the authors have critically read the manuscript and approved the submitted version.

## FUNDING INFORMATION

This work was supported by Changping Laboratory (2021B‐01‐01); the National Natural Science Foundation of China (81301032, 62201023); the State Key Laboratory for Infectious Disease Prevention and Control, China CDC (2020SKLID311); the Open Project of State Key Laboratory of Infectious Disease Prevention and Control (2020SKLID311); and the China Postdoctoral Science Foundation (2022M720529).

## CONFLICT OF INTEREST STATEMENT

None.

## CONSENT

All participants or their designees provided informed consent.

## Supporting information


Data S1:

## Data Availability

The data that support the findings of this study are available from the corresponding author upon reasonable request.
