# Supplementary material for: Personalized brain MRI revealed distinct functional and anatomical disruptions in Creutzfeldt‐Jakob disease and Alzheimer's disease
Source: CNS Neurosci Ther. 2023 Aug 14;30(2):e14404. doi: 10.1111/cns.14404 (PMC10848072; doi:10.1111/cns.14404)
Supplement: Supplementary file 1 — Data S1: [file CNS-30-e14404-s001.docx]

**Supplementary Materials**

**Supplementary Methods**

***1. Preprocessing***

Briefly, the following steps were performed: (1) slice timing correction (SPM2; Wellcome Department of Cognitive Neurology, London, UK), (2) head motion correction with FSL 5.0.4 (http://surfer.nmr.mgh.harvard.edu/fswiki/FsFast), (3) normalization of global mean signal intensity across runs, (4) bandpass filtering (0.01-0.08 Hz), and (5) nuisance variables regression, including 6 motion parameters, white matter signal, ventricular signal, whole brain signal, and their first-order temporal derivatives.

Structural data was processed using the FreeSurfer version 5.3.0 software package (<http://surfer.nmr.mgh.harvard.edu>). Surface mesh representations of the cortical cortex were reconstructed from structural images and registered to a common spherical coordinate system (fsaverage6). FMRI images were linearly aligned to the participant’s structural images using boundary-based registration ^1^. Next, a 6-mm ﻿full-width half-maximum smoothing kernel was applied to the fMRI data.

***2. Individualized Parcellation***

A coarse-to-fine strategy was performed to generate the fine-grained parcellation ^2^. Briefly, the cerebral cortex was split into five regions—frontal, temporal, parietal, occipital, and central regions—according to ﻿the Desikan-Killiany atlas ^3^, and then each region was segmented into multiple subareas using a *k*-means clustering approach on the basis of the population-level rsFC profile. The rsFC profile was estimated by calculating the Pearson correlation between the time series of each vertex in FreeSurfer fsaverage6 space and the 1,175 ROIs ^4^.

Specifically, a fine-grained ROI was assigned to the network that presented the highest Dice overlap coefficient with said ROI. We also further grouped the eight networks into either the primary cortex, which included the VIS and MOT, or the association cortex, which included the dATN, vATN, LMB, FPN, LAN, DMN. The canonical large-scale functional networks were adjusted from Yeo’s 7-network parcellation, which was also derived from the GSP dataset ^4^.

**Supplementary Results**

***1. Additional analyses***

We performed additional analyses to examine whether the anatomical and functional changes are related to the progression of the disease. For the five CJD patients with two scans, we correlated the time interval between the two scans with cortical thinning (Fig. S5 in the supplement). There was no significant correlation with cortical thinning in the primary cortex (Pearson’s correlation, *r* = -0.13, *p* = .84, Fig. S5a in the supplement) or the association cortex (Pearson’s correlation, *r* = -0.36, *p* = .54, Fig. S5b in the supplement). Lastly, we correlated the time interval between scans with rsFC strength decrease. There was a marginally significant correlation in the primary cortex (Pearson’s correlation, *r* = 0.86, *p* = .06, Fig. S5c in the supplement), but no significant correlation in the association cortex (Pearson’s correlation, *r* = 0.70, *p* = .19, Fig. S5d in the supplement). Despite the small sample size, the relatively high correlation values indicate that rsFC may be a potential biomarker for monitoring the rapid progression of CJD.

**References**

1 Greve DN, Fischl B (2009) Accurate and robust brain image alignment using boundary-based registration. Neuroimage 48:63-72

2 Lebois LAM, Li M, Baker JT et al (2021) Large-Scale Functional Brain Network Architecture Changes Associated With Trauma-Related Dissociation. Am J Psychiatry 178:165-173

3 Desikan RS, Segonne F, Fischl B et al (2006) An automated labeling system for subdividing the human cerebral cortex on MRI scans into gyral based regions of interest. Neuroimage 31:968-980

4 Yeo BT, Krienen FM, Sepulcre J et al (2011) The organization of the human cerebral cortex estimated by intrinsic functional connectivity. J Neurophysiol 106:1125-1165


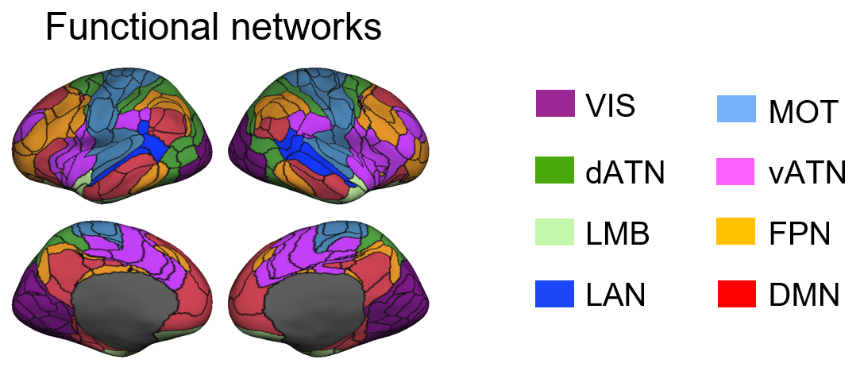


**Figure S1. The 213 functional ROIs were assigned to eight canonical large-scale functional networks indicated by different colors.** VIS and MOT belong to the primary cortex, while the other networks belong to the association cortex.


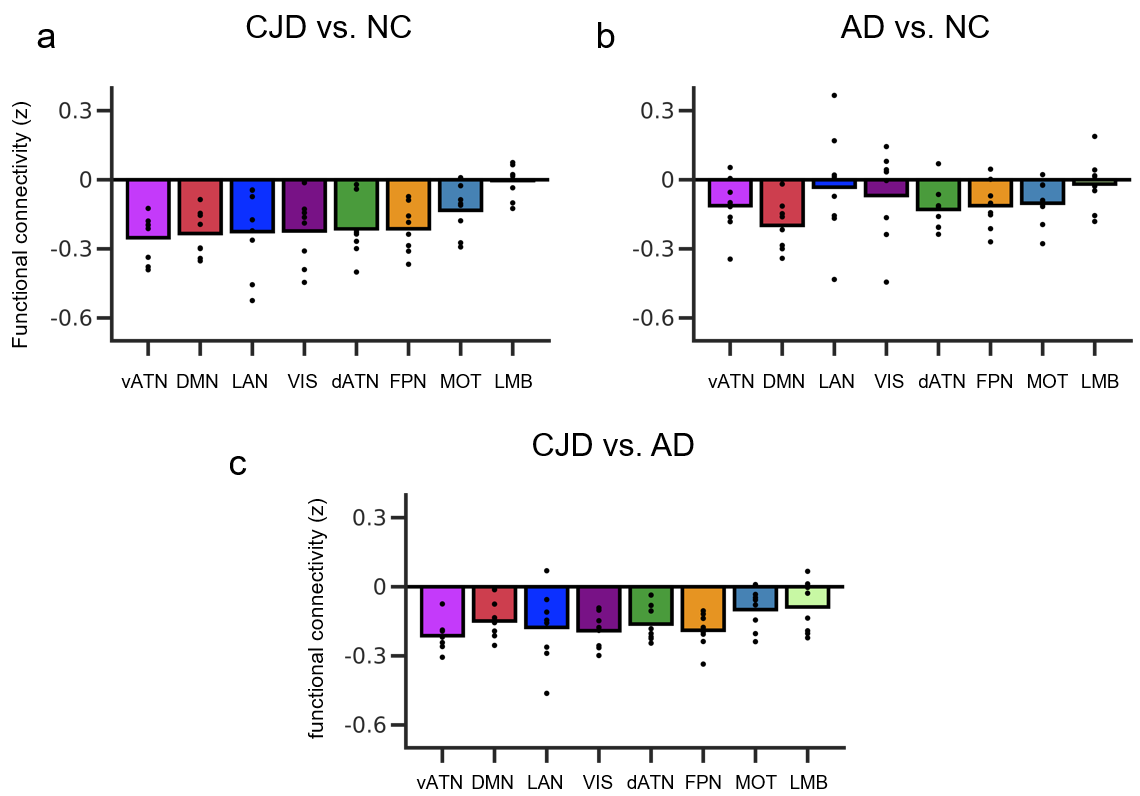


**Figure S2. CJD shows reductions in rsFC in all networks compared to AD patients.** (**a**) Reductions of rsFC was estimated by contrast the rsFC strength of CJD and NC groups for each of the 8 large-scale networks. Each black dot represents the rsFC reduction for one CJD patient compared to the corresponding NC. All networks show rsFC reduction except for the LMB network. (**b**) RsFC strengths of AD patients in each network is compared with NC group. (**c**) We compared the rsFC strength of CJD and AD groups. The contrast map shows that rsFC of CJD is weaker than that of AD in all networks.


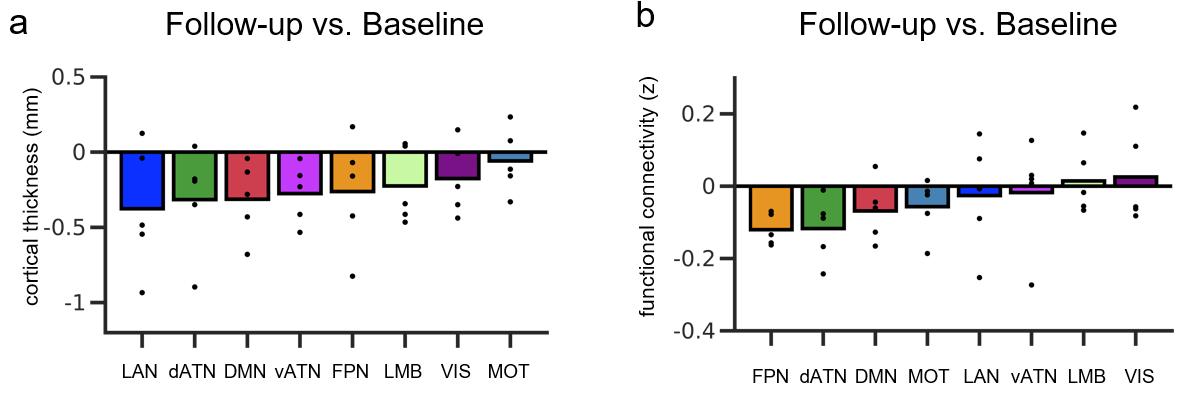


**Figure S3. Cortical thinning and disruption of rsFC at the follow-up of CJD compared to those of the baseline.** (**a**) Cortical thinning (follow-up – baseline of CJD) was estimated within each of the 8 large-scale networks. Each black dot represents cortical thinning for one CJD patient. Most networks show cortical atrophy at follow-up. (**b**) RsFC strength at follow-up shows decrement mainly in FPN, dATN and DMN networks.


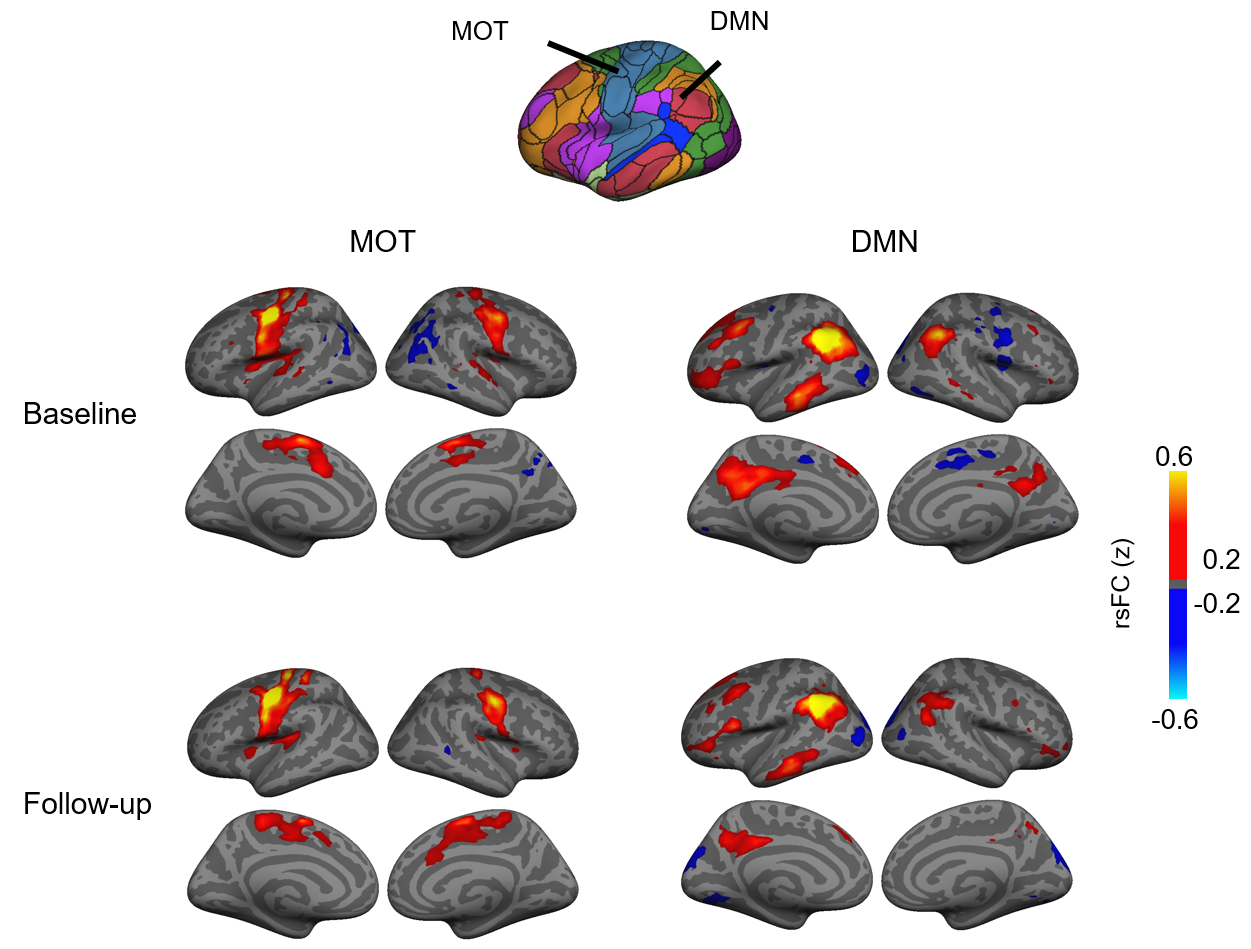


**Figure S4. Average ROI connectivity maps of CJD patients at baseline and at follow-up.** We compared the average functional connectivity maps of MOT and DMN networks. Slight difference is shown in the ROI connectivity of MOT network at follow-up. RsFC connectivity of DMN seed is weaker at follow-up in both hemispheres.


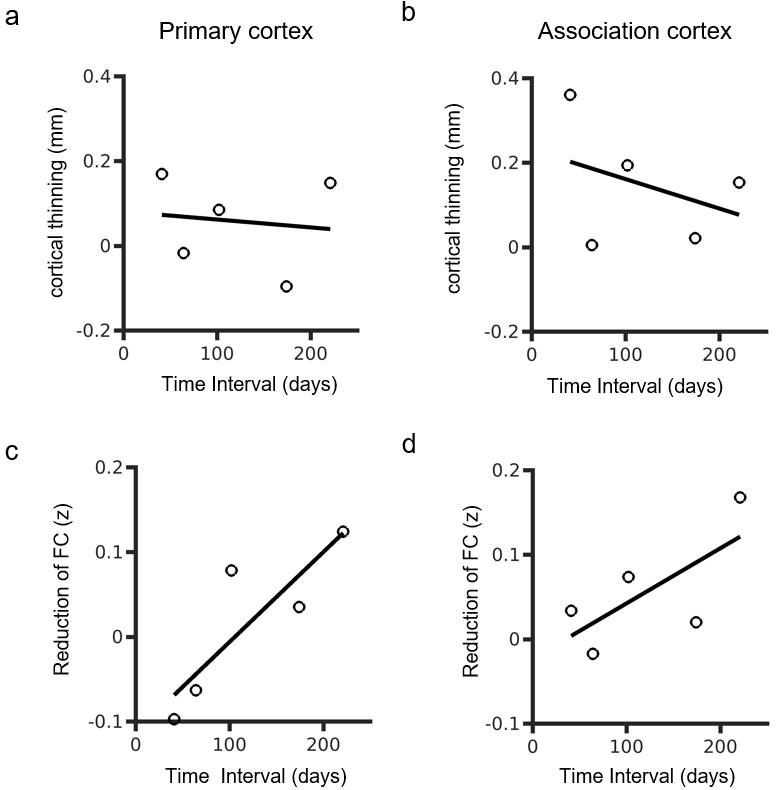


**Figure S5. Correlation between time intervals and anatomical and functional disruptions.** (**a**) There is no significant correlation between the time interval and cortical thinning in the primary cortex (*r* = -0.13, *p* = .84). (**b**) No significant correlation is shown between the time interval and the cortical thinning in the association cortex (*r* = -0.36, *p* = .54). (**c**) RsFC strength reduction in the primary cortex is significantly associated with the time interval (*r* = 0.86, *p* = .05). (**d**) There is no significant correlation between the time interval and the rsFC strength reduction in the association cortex (*r* = 0.70, *p* = .19).


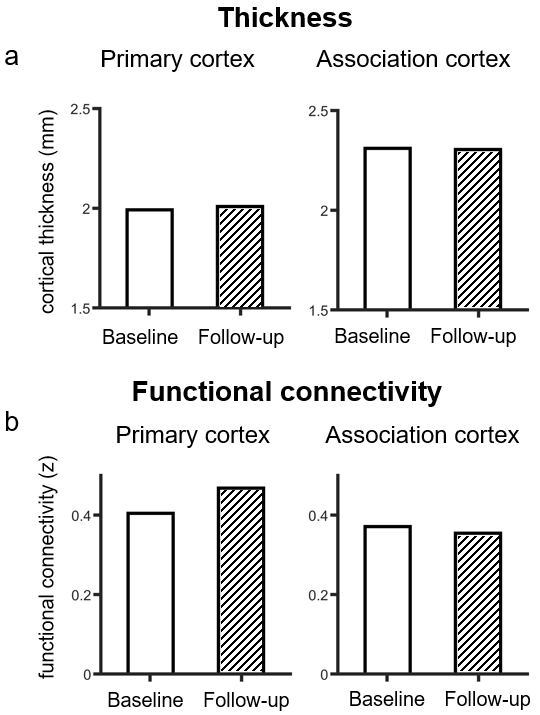


**Figure S6. The progressive of anatomical and functional changes of patient CJD04.** (a) Cortical thinning (follow-up – baseline) increased by 0.83% in primary cortex, and decreased by 0.24% in association cortex. (b) Functional connectivity increased by 15.56% in primary cortex, and decreased by 4.58% in association cortex.
